# Potential International Approaches to Ownership/Control of Human Genetic Resources

**DOI:** 10.1007/s10728-015-0300-4

**Published:** 2015-08-22

**Authors:** Catherine Rhodes

**Affiliations:** Institute for Science, Ethics and Innovation, 3.614 Stopford Building, Faculty of Life Sciences, University of Manchester, Oxford Road, Manchester, M13 9PL UK

**Keywords:** International governance, Human genetic resources, Common heritage of mankind, Approaches to genetic resources ownership, Genetic resources governance

## Abstract

In its governance activities for genetic resources, the international community has adopted various approaches to their ownership, including: free access; common heritage of mankind; intellectual property rights; and state sovereign rights. They have also created systems which combine elements of these approaches. While governance of plant and animal genetic resources is well-established internationally, there has not yet been a clear approach selected for human genetic resources. Based on assessment of the goals which international governance of human genetic resources ought to serve, and the implications for how they will be accessed and utilised, it is argued that common heritage of mankind will be the most appropriate approach to adopt to their ownership/control. It does this with the aim of stimulating discussion in this area and providing a starting point for deeper consideration of how a common heritage of mankind, or similar, regime for human genetic resources would function and be implemented.

## Introduction

Scientific work utilising human genetic resources has great potential for contributing to addressing major global health challenges. The approach adopted to their ownership in international governance will have significant implications for how they are accessed and used, who participates in this work, the direction it takes and who benefits from it. This paper outlines the different approaches to genetic resources ownership/control that have been taken by the international community, before assessing which will be the most appropriate approach to adopt toward governance of human genetic resources, which—in the absence of an agreed international approach—are likely to fall under free access, which tends to privilege the interests of private companies and scientifically and technologically advanced states, and is therefore inappropriate to serve global health needs.

## Use of Terms

### Genetic Resources

Genetic resources have been defined in international law as “genetic material of actual or potential value”, with genetic material being “any material of plant, animal, microbial or other origin containing functional units of heredity” (Convention on Biological Diversity, Article 2 [3]). The term can also be understood to include associated data. There is still some uncertainty about whether the term genetic resources covers ‘derivatives’, defined in Article 2 of the Nagoya Protocol to the Convention on Biological Diversity as “a naturally occurring biochemical compound resulting from the genetic expression or metabolism of biological or genetic resources, even if it does not contain functional units of heredity” [4] (see for example section 2.4.1 in [15, 28]). Traditional knowledge associated with certain genetic resources also receives particular protection, but is not covered in any detail in this paper.

### Human Genetic Resources

In line with this definition, human genetic resources are understood to be human genetic material, containing functional units of heredity, with actual or potential value, and associated data.

### International Community

Where the term international community is used in this paper, it primarily refers to states and the international organisations of which they are members.

### International Governance

The term international governance refers to rules, norms, institutions, procedures and mechanisms which govern the behaviour of states and other international actors in the absence of supranational government. Significant among these in the genetic resources area are international organisations, treaties, standards, guidelines and codes, and information exchange, reporting and surveillance mechanisms (examples are shown in Table 1). A useful, complementary definition of international governance is provided by Finkelstein [10, p. 368]: “any purposeful activity intended to ‘control’ or influence someone else that either occurs in the arena occupied by nations or, occurring at other levels, projects influence into that arena”. This paper focuses on elements of governance that are potentially universal—that is open to all states to subscribe to, without any limitations on e.g. geographic or economic grounds.

**Table 1:** Selected examples of international governance mechanisms for genetic resources

International organisations	Treaties	Standards/guidelines/codes/declarations	Reporting and surveillance mechanisms/expert networks
Food and Agriculture Organisation United Nations Educational, Scientific and Cultural Organisation World Animal Health Organisation World Health Organisation World Intellectual Property Organisation World Trade Organisation	Biological Weapons Convention Convention on Biodiversity International Plant Protection Convention International Treaty on Plant Genetic Resources for Food and Agriculture Nagoya Protocol on Access to Genetic Resources and the Fair and Equitable Sharing of the Benefits Arising from their Utilisation	Aquatic Animal Health Code Terrestrial Animal Health Code Laboratory Biosafety Manual Laboratory Biosecurity Guidance Universal Declaration on the Human Genome and Human Rights United Nations Declaration on the Rights of Indigenous Peoples	Emergency Prevention and Response System—Food Safety Global Influenza Surveillance and Response System World Animal Health Information Service World Information and Early Warning System for Plant Genetic Resources for Food and Agriculture

## Governing Genetic Resources at the International Level

There have been international efforts to govern genetic resources for over 60 years. At first these focused on exchanges of plant genetic resources, and governance is most developed in this area. Many of the current rules on genetic resources—while having wider scope—are designed around historical patterns of exploitation and exchange associated with plants. Genetic resources governance is least developed for the areas of human and microbial genetic resources. For human genetic resources it is currently limited to three declarations of principles (not legally-binding on states) and concentrated within the United Nations Educational, Scientific and Cultural Organisation. Human genetic resources could justifiably fall within the remit of the World Health Organisation in regard to their (primary) utility in the understanding of human disease. The Organisation for Economic Cooperation and Development has also published some guidelines relating to human genetic databanks—I do not consider these to fall in the scope of international rules, which for the purposes of this analysis, I limit to those rules that are potentially universal—however, they provide a useful indicator of the direction the international community may take as it develops rules in this area.

Genetic resources governance has expanded massively in scope from its original concern with facilitating exchange of plant genetic material. As well as covering collection, exchange and banking of genetic resources, it now covers several other issues including: conservation; protection of human rights; protection of human, animal and plant health; food security; climate change adaptation and mitigation; finance, funding and capacity building (particularly in science and technology); access and benefit-sharing; and ownership and control rights. Approaches to genetic resources ownership and control are of central interest in this paper.

## International Approaches to Ownership/Control of Genetic Resources

There are, broadly, five approaches^1^ to ownership/control^2^ of genetic resources that are used by the international community: free access; state sovereign rights; intellectual property rights; common heritage of mankind; and mixed systems. Their general use is outlined next, before they are discussed in the more specific context of human genetic resources governance.

### Free Access

Where a free access approach is applied to genetic resources, anyone is free to access them, to use them as they choose, and to subsequently claim proprietary rights over them (excluding others from access/use if they choose to do so). This was the dominant international approach to genetic resources governance prior to the application of state sovereign rights in the early 1990s. It is a default position where no rules have been established, and still operates for certain genetic resources, such as those located in some areas defined as ‘beyond national jurisdiction’—for example marine genetic resources in the high seas and seabed beyond state jurisdiction. Free access is controversial because it tends to favour those who have particular knowledge and expertise, and the financial and technological means to access and exploit the resources [1]—which are primarily concentrated in developed countries. Free access therefore tends to have the effect of concentrating benefits of research on and use of genetic resources in a limited number of individuals, groups and states, regardless of where they are sourced from.

### State Sovereign Rights

State sovereignty has long been accepted to include rights to territorial integrity and control over resources within that territory—particularly mineral resources. This is, for example, repeatedly stated in resolutions of the United Nations General Assembly, such as Resolution 3016(XXVII) ‘Permanent sovereignty over natural resources of developing countries’, which: “Reaffirms the right of States to permanent sovereignty over all their natural resources, on land within their international boundaries” [27].

State sovereign rights have been applied to genetic resources by the international community since the early 1990s, and are now the dominant international approach to genetic resources ownership/control. While not yet explicitly extended to human genetic resources, sovereign rights were extended to viral genetic resources in 2011 on quite spurious grounds [18], and so while their extension to human genetic resources seems inappropriate, it is not implausible.

Statements of state sovereignty over genetic resources in international law generally use similar wording to that found in the Convention on Biological Diversity (CBD), but are often specific to particular sub-sets/types of genetic resources (such as plant genetic resources, or influenza genetic resources). Based on recognition of “the sovereign rights of States over their natural resources” Article 15.1 of the CBD states that: “the authority to determine access to genetic resources rests with the national governments and is subject to national legislation”.

Within international agreements that adopt a sovereign rights approach, access to genetic resources is made subject to the prior informed consent of the provider state, which can negotiate conditions on the sharing of any benefits that arise from their subsequent exploitation. This is, for example, set out in Articles 5 and 6 of the Nagoya Protocol (to the Convention on Biological Diversity) on Access to Genetic Resources and the Fair and Equitable Sharing of the Benefits Arising from their Utilisation:

Article 5—Fair and Equitable Benefit-sharing

…benefits arising from the utilization of genetic resources as well as subsequent applications and commercialization shall be shared in a fair and equitable way with the [State] Party providing such resources… Such sharing shall be upon mutually agreed terms.

Article 6—Access to Genetic Resources

1. In the exercise of sovereign rights over natural resources, and subject to domestic access and benefit-sharing legislation or regulatory requirements, access to genetic resources for their utilization shall be subject to the prior informed consent of the [State] Party providing such resources…

3. Pursuant to paragraph 1 above, each Party requiring prior informed consent shall take the necessary legislative, administrative or policy measures, as appropriate, to:…

(g) Establish clear rules and procedures for requiring and establishing mutually agreed terms. Such terms shall be set out in writing and may include, inter alia: …

(ii) Terms on benefit-sharing, including in relation to intellectual property rights;

Use of a sovereign rights approach does not necessarily preclude subsequent claims of intellectual property rights being made by the user—it will depend on the terms agreed between the user and provider state.

Access and benefit-sharing arrangements based on state sovereignty are generally made through use of standard material transfer agreements which form a contract between the provider and the user.^3^ Recommendations and/or requirements for the content of these agreements are generally set out in the relevant treaty, and model contracts are usually provided by the relevant international organisation.

Often, within provisions on access and benefit-sharing, there is incorporation of respect for the rights of farmers, local and indigenous communities over genetic resources and associated knowledge, which they traditionally hold or have played a key role in developing. States are expected, for example, to ensure such groups are involved in consent processes and benefit-sharing negotiations.

### Intellectual Property Rights

There are two main forms of intellectual property right which can be claimed over genetic resources—patents and plant variety rights. The latter obviously do not apply to human genetic resources; there is considerable controversy over whether patents should apply to them. Patents are increasingly being claimed over a range of genetic resources. The appropriateness of this is a subject of intense debate, both because of disputes over the extent to which genetic resources fulfil criteria of novelty and inventiveness required for grant of patents,^4^ and also because it is seen as frequently being unfair to provider states and communities—their contributions are rarely acknowledged, their knowledge is often absent from searches for prior art, and they may be excluded from benefits arising from commercial exploitation. This is for example a major source of contention in the World Trade Organisation’s TRIPS Council^5^ (the committee which oversees the review of the Trade Related Aspects of Intellectual Property Rights Agreement, which sets international minimum standards of intellectual property protection). The subject is considered under combined agenda items on: Review of the Provisions of Article 27.3 (b); Relationship between the TRIPS Agreement and the Convention on Biological Diversity; and Protection of Traditional Knowledge and Folklore, at each regular meeting of the TRIPS Council. South Africa, for example, summarised the problem areas in its statement to the Council in November 2012 [34, pgh. 33], noting “three fundamental conflicts between the Convention on Biological Diversity and TRIPS Agreement”: first, that TRIPS overlooks state sovereign rights over genetic resources as provided by the CBD; second, that TRIPS negates states’ legal authority to determine benefit-sharing from commercial use of genetic resources; and third, that TRIPS does not incorporate requirements for prior informed consent within patent applications.

Very limited progress has been made on the issues over the past 15 years, but discussions remain on-going. Reform suggestions made, for example in the World Trade Organisation’s Trade Negotiations Committee [35, pghs. 4–6, and 36] and through the World Intellectual Property Organisation’s Intergovernmental Committee on Intellectual Property Rights, Genetic Resources, Traditional Knowledge and Folklore [29, 30], have included: incorporating traditional knowledge in prior art searches; requiring disclosure of origin of genetic resources or traditional knowledge used in patent applications; and requiring evidence of compliance with access and benefit-sharing rules in order to be granted a patent.

### Common Heritage of Mankind

The concept of common heritage of mankind^6^ began to be articulated internationally during the late 1960s and has been used by developing states in efforts to shape international law relating to common areas (there are four ‘global commons’ managed by the international community: Antarctica; the high seas and seabed beyond areas of national jurisdiction; the atmosphere; and outer space). While developed in relation to common areas, the common heritage of mankind approach can be adapted to common resources. Joyner [12, pp. 191–2] conceptualised common heritage of mankind^7^ for common areas as being based on five main elements:

1. not… subject to appropriation of any kind, either public or private, national or corporate… owned by no one, though hypothetically managed by everyone. Sovereignty would be absent, as would all its legal attributes and ramifications… legally the entire area would be administered by the international community.

2. all people would be expected to share in the management of a common space area… States or national governments would be precluded from this legal function, save as the representative agents of all mankind… universal popular interests would assume priority, and thereby supply the foundation for any administrative decisions made affecting the region.

3. if natural resources were exploited… any economic benefits derived from those efforts would be shared internationally. Under a CHM [Common Heritage of Mankind] regime, agencies engaged in commercial profit or private gain would be deemed inappropriate, unless they operated to enhance the common benefit of all mankind.

4. use of the area must be limited to exclusively peaceful purposes

5. scientific research… would be freely and openly permissible, so long as the environment of the common space area was in no way physically threatened or ecologically impaired. All research results would be made available as soon as possible to anyone who genuinely expressed interest in them. Under a CHM regime, scientific research would be conducted to benefit all peoples, not merely the State or government which sponsored the research. Furthermore, the scientific fruits of such research would be freely and publicly exchanged in the hope of fostering greater scientific co-operation and more extensive knowledge about the region.

Joyner also noted that an international authority would be needed in order to effectively manage such areas as common heritage of mankind, and that the authority would need to fulfil various legal functions such as “distributing users’ rights and economic benefits… and facilitating the settlement of disputes” [12, p. 194].

Applying these elements to genetic resources would mean that: resources would not be subject to appropriation and would be managed in line with universal interests; any economic (or other) benefits arising from their exploitation would be shared internationally; their use would be limited to exclusively peaceful purposes^8^; and scientific research using genetic resources would be conducted for the benefit of all. Elements of the common heritage approach are covered in more detail below, where it is applied specifically to human genetic resources.

Common heritage of mankind has little current use as an approach to genetic resources governance, but it is promoted by many developing countries for marine genetic resources in areas beyond national jurisdiction. Currently, these resources are subject to free access, which is advantaging rich, technologically advanced states and commercial enterprises which are able to extract the resources and exploit them. The United Nations Convention on the Law of the Sea (UNCLOS) places the seabed and ocean floor and the mineral resources found there under the common heritage of mankind [26, Article 136], and while developing countries argue that this should apply to genetic resources as well, developed countries argue that they instead fall under the freedom of the high seas [2].

There was also an unsuccessful attempt to have common heritage of mankind applied to plant genetic resources in the early 1980s, within the Food and Agriculture Organisation’s International Undertaking on Plant Genetic Resources, which originally stated that: “This Undertaking is based on the universally accepted principle that plant genetic resources are a heritage of mankind and consequently should be available without restriction” [6, Article 1]. The Undertaking received little support from developed states because of its inclusion of newly developed varieties and breeding lines within the scope of common heritage, and was amended in 1991 to give priority to the sovereign rights of states: “Recognizing that:—the concept of mankind’s heritage, as applied in the International Undertaking on Plant Genetic Resources, is subject to the sovereignty of the states over their plant genetic resources…” [7].

### Mixed Systems

The international community has established two systems for genetic resources which combine elements of state sovereignty and centralised access and/or benefit-sharing mechanisms, with some allowance for claims to intellectual property rights. The first of these is the Multilateral System of Access and Benefit-Sharing created by the International Treaty on Plant Genetic Resources, which became operational in October 2007 [8]. It covers a list of food and forage crops determined to be particularly important to food security. States agree to exercise their sovereign rights through the System, using it to facilitate access to their listed plant genetic resources [9, Article 12.3 (d) and (f)]. They are encouraged to do so through use of standard material transfer agreements, which should include provisions on benefit-sharing—including benefits such as information exchange, technology transfer, capacity building, and monetary benefits from commercialisation [9, Article 12.4 and Article 13].

The second example is the Pandemic Influenza Preparedness Framework adopted by the World Health Organisation in 2011. This focuses on influenza viruses with human pandemic potential and centralises the sharing of influenza viral genetic resources for international collaborative research efforts within its Global Influenza Surveillance and Response System (GISRS) and between the System and external entities (generally pharmaceutical companies and vaccine manufacturers). It also provides centralised benefit-sharing systems including vaccine and anti-viral stockpiles, which can be distributed during pandemics. State sovereign rights over their influenza viral genetic resources are recognised alongside the importance of sharing them for global public health efforts. Sharing of biological materials under the Framework is done using standard material transfer agreements in two forms: one between entities within GISRS—Standard Material Transfer Agreement 1; the other between the World Health Organisation and entities external to the GISRS—Standard Material Transfer Agreement 2 [32, Annex 1 and Annex 2]. The state affected by an influenza outbreak with human pandemic potential is expected to submit samples to the GISRS in a timely manner [32, pgh. 5.1.1]. The state may also provide samples directly to companies, provided GISRS access is prioritised [32, pgh. 5.1.4]. Subsequent claims of intellectual property rights by such companies does not seem to be precluded, however the implications of this for work within GISRS are unclear.

## Governing Human Genetic Resources at the International Level

Applying state sovereign rights to human genetic resources would be problematic and has not entered state practice. It would, for example, imply that the state had a right to determine access to the genetic material of people within its jurisdiction, and to be party to any benefits accruing from their utilisation. Currently, there is no established international approach to the ownership of human genetic resources. On a practical basis this means that free access is—by default—likely to apply. As outlined above, this means that anyone can access such resources, utilise them as they choose,^9^ and claim proprietary rights over them. Free access situations tend to privilege those with advanced scientific and technological capacity, and may well lead to concentration of the benefits of human genetic research in developed states and commercial entities. Claims of intellectual property rights can be detrimental to the achievement of important global goals—in this case advances in human health, and increase the costs of participation in scientific research. Concern about this situation was expressed at length in the World Health Organisation [31] report *Genomics and World Health*. It noted, for example, that trends in DNA patenting (of human and pathogenic genetic material) mean that “future profits and resource flows in genomics will be concentrated in the developed economies in general, and in the United States in particular” (p. 128) and concluded that “the current position regarding DNA patenting is retarding rather than stimulating both scientific and economic progress” (p. 138). These patenting trends have continued—a 2013 study found that approximately 41 % of human genes are covered by patents [19].

There are ongoing disputes about whether proprietary rights over human genetic resources are valid or appropriate. As well as the broader arguments raised against the patenting of genetic resources in general (outlined earlier), additional arguments have been put forward in opposition to patenting of human genetic resources—for example in regard to the added expense this might bring to the cost of diagnostics—the Myriad case being an example of this (see [14] for an overview of the case and its implications), the impacts it can have on research with major public health benefits, and concerns over the commodification of life (see for example [11, 13]).

Given these problems with use of state sovereign rights, free access and intellectual property rights approaches to human genetic resources governance, a common heritage approach may be the most suitable alternative.

### The Appropriateness of a Common Heritage Approach to Human Genetic Resources

This section considers whether a common heritage approach to governance of human genetic resources is appropriate both in terms of the goals that such governance ought to be achieving and the principles articulated by the international community about the status and appropriate management of human genetic material. Joyner’s five elements of common heritage of mankind would apply to human genetic resources as follows:

1. Human genetic resources could not be owned and/or appropriated by anyone, nor could they be subject to sovereign rights. They would be administered by the international community on behalf of all mankind, with attention being given to the priority needs of developing countries where necessary.^10^

2. The international community would share responsibility for the resources and would manage them on the basis of universal interests.

3. Benefits from the exploitation of human genetic resources would be shared internationally on the basis of common global interests and with attention to the needs of developing countries, and “agencies engaged in commercial profit or private gain would be deemed inappropriate, unless they operated to enhance the common benefit of all mankind” [12, p. 192].

4. The use of human genetic resources would be limited to peaceful purposes.

5. Scientific research on human genetic resources would be freely and openly permitted (provided it is in compliance with other international and applicable domestic laws—for example on the protection of research subjects). The results of such research would rapidly be made freely available to anyone with a genuine interest. Scientific research would be directed to the benefit of all peoples and the fruits of such research would be freely and publicly exchanged.

(Adapted from [17], p. 235).

### Goals of Human Genetic Resources Governance

Goal: achieving advances in human health (e.g. through better understanding, diagnosis, prevention and treatment of disease).

The primary utility of human genetic resources is for research into human disease processes and their interactions with environmental factors. There is a common global interest in efforts for disease control and for scientific and medical work which supports these; within international governance a focus on global priority health needs is appropriate. A common heritage approach will direct work on human genetic resources towards universal interests (under point 2) and so fits well with this goal.

This is supported by the Universal Declaration on the Human Genome^11^ and Human Rights [23], the International Declaration on Human Genetic Data [24], and the Universal Declaration on Bioethics and Human Rights [25], which all incorporate the principle that research involving human genetic resources should benefit humanity as a whole, and particularly in relation to health goals: “The applications of research… concerning the human genome, shall seek to offer relief from suffering and improve the health of individuals and humankind as a whole” [23, Article 12].

The importance of this has been recognised in scientists’ reflections on progress within the Human Genome Project—John Sulston, for example, noting that:

Science is international, and its benefits ought to be international as well—at least with regard to such a basic human need as healthcare. Yet this is far from the case. Most biomedical research is aimed at the rich markets, and most of the human disease burden is, so far, untouched by technological progress. We urgently need to do better—not just for the sake of our common humanity but for all our futures, as a divided world is unstable and dangerous [21, p. 14].

Goal: capacity building and reduction of inequalities in health and medical research and healthcare systems.

There is also a common interest in the management and use of human genetic resources contributing to development and reduction in inequalities. To make responses to health threats sustainable, substantial capacity building is needed in order to reduce inequalities in e.g. health infrastructure, and access to medical care and medicines (this has been sharply illustrated during the current Ebola outbreak—see, for example, [33]).

There also need to be reductions of inequalities in scientific and technological capacities between states. These currently have a major influence on what research gets done, how it is directed, who participates, and who benefits from it. Measures to facilitate this will include extensive capacity building efforts of various kinds to increase participation in research using human genetic resources (e.g. in infrastructure, education, science, technology, knowledge, expertise, research facilities and equipment, regulation and administration).

A common heritage approach will require equitable sharing of economic and other benefits of work with human genetic resources internationally, including both increased knowledge and the improved products that might result from it (fitting with points 3 and 5 of Joyner’s approach). Such benefit-sharing can support the needed capacity-building efforts and assist reduction of inequalities in the areas of health and scientific and technological capacities.

The international authority that would be associated with management of human genetic resources under a common heritage approach, could help to direct funds (derived from centralised benefit-sharing mechanisms) toward capacity-building, and take on other related functions, in a similar way to the governing body for the UN Convention on the Law of the Sea (known as ‘the Authority’). The Authority is assigned various responsibilities relating to the conduct, coordination and oversight of activities—such as marine scientific research—that take place within the ‘Area’ (which is beyond national jurisdiction and to which common heritage of mankind applies). These include:

- Promoting scientific research for the benefit of mankind as a whole, with particular consideration to the needs and interests of developing states (Articles 140 and 143).
- Promoting international cooperation in such research including capacity-building for developing states (Article 143.3).
- Facilitating the exchange and dissemination of scientific knowledge, data and information, and promoting provision of scientific and technical assistance and technology transfer, so that all can benefit from research in the Area (Articles 144, 202, 244.2 and 266).
- Promoting effective participation in activities by developing states (Article 148).

Such capacity-building efforts are promoted extensively in the three UNESCO declarations, for example:

(a) … benefits resulting from the use of human genetic data, human proteomic data or biological samples collected for medical and scientific research should be shared with the society as a whole and the international community. In giving effect to this principle, benefits may take any of the following forms:

(i) special assistance to the persons and groups that have taken part in the research;

(ii) access to medical care;

(iii) provision of new diagnostics, facilities for new treatments or drugs stemming from the research;

(iv) support for health services;

(v) capacity-building facilities for research purposes;

(vi) development and strengthening of the capacity of developing countries to collect and process human genetic data, taking into consideration their specific problems [24, Article 19].

(a) In the framework of international cooperation with developing countries, states should seek to encourage measures enabling: … (ii) the capacity of developing countries to carry out research on human biology and genetics, taking into consideration their specific problems, to be developed and strengthened; (iii) developing countries to benefit from the achievements of scientific and technological research so that their use in favour of economic and social progress can be to the benefit of all; (iv) the free exchange of scientific knowledge and information in the areas of biology, genetics and medicine to be promoted [23, Article 19].

However, the statement in Article 4 of the Universal Declaration on the Human Genome and Human Rights that “the human genome in its natural state shall not give rise to financial gains” [23] is problematic: ‘natural state’ is subject to varying interpretations and this is one reason for controversies over patentability of genetic resources (i.e. whether they are inventions or discoveries); and to fit with the common heritage approach it will need to extend beyond this to ‘worked’ resources, associated data and products and processes emerging from research. It would need to be amended, for example, to state that financial gains from work on the human genome, even in its natural state, are acceptable as long as they are shared internationally and result from work which is for common benefit. Such an amendment reflects Joyner’s approach at point 3 and is consistent with the extracts from the Universal Declaration on the Human Genome and Human Rights and the International Declaration on Human Genetic Data quoted above.

Goal: effective collection and exchange mechanisms and procedures to facilitate scientific research.

Goal: interoperability between collections (e.g. in the types of data collected and how it is recorded, in software systems, and in standards relating to e.g. confidentiality and anonymity).

As well as benefit-sharing and capacity building, the international authority would have other administrative, regulatory and oversight functions in relation to the management of human genetic resources. These could include the development of standards for collection, storage and exchange (the FAO has undertaken similar tasks for plant genetic resources^12^). This would, for example, boost collaboration by opening “up a wider range of samples and improve applicability to a broad range of contexts” in health-related research on human genetic resources, and “provide clarity on roles and responsibilities and simplify transactions” between genebanks and research institutions, helping “to reduce restrictions on exchange that stem from uncertainty about whether the standards required by the provider… can or will be met by the recipient” [17, p. 68].

The benefits of facilitating international collaborations in human genetic and genomic research projects were outlined by Collins in [5]:

A second lesson from the HGP [Human Genome Project] was the importance of international participation. Although the complexities of organizing collaborations and conference calls across multiple time zones can present challenges, the arguments in favour of maximum worldwide participation are strong… Furthermore, the human genome is our shared inheritance; it is therefore highly appropriate and desirable to have scientists of many different languages and cultures working together on such projects. Finally, the opportunity to include participation by scientists in countries where the infrastructure for biomedical research is not yet optimally developed can provide a valuable ‘on ramp’ for such individuals to acquire technology, funding and public recognition within their own countries.

The UN Convention on the Law of the Sea’s provisions on the application of common heritage of mankind contain clauses regarding the conduct of scientific research that, for example, relate to protection of the environment and sustainable management. Common heritage applied to human genetic resources could include protective measures for human participants in research and some limitations on access to data and materials so that, e.g. confidentiality and informed consent can be preserved. This would be achieved through standard setting functions assigned to the associated international authority and this would enable interoperability to be maintained, alongside assurance that compatible protective measures are in place.

Goal: facilitation of scientific research on human genetic resources.

Scientific practice—for example in large-scale, international collaborative genome projects^13^—indicates that releasing information into the public domain contributes significantly to rapid developments in the field of human genomics (see for example [5, 21]).

A common heritage approach would freely and openly permit scientific research on human genetic resources, with research results being made available as soon as possible to all with a genuine interest. Research on human genetic resources would be conducted for the benefit of all peoples, and its ‘fruits’ would be freely and publicly exchanged. If the sharing in the ‘fruits of such research’ involves (as it seems to) more than the sharing of results, and extends for example to access to products and processes which utilise human genetic resources, then there will also need to be centralised funding schemes that enable some form of cost-sharing.

This element of a common heritage approach is also supported by provisions within the three UNESCO Declarations. The Universal Declaration on the Human Genome and Human Rights, for example, states that: “states should take appropriate measures to foster the intellectual and material conditions favourable to freedom in the conduct of research on the human genome”; and “States should make every effort… to continue fostering the international dissemination of scientific knowledge concerning the human genome, human diversity and genetic research and, in that regard, to foster scientific and cultural cooperation, particularly between industrialized and developing countries” [23, Articles 14 and 18]. Very similar provisions appear in the International Declaration on Human Genetic Data and the Universal Declaration on Bioethics and Human Rights.

Goal: an internationally agreed approach to their management, to facilitate these other activities.

Given these goals, it is clear that taking the step of achieving an internationally agreed approach to the management of human genetic resources is necessary in order to optimise the contribution of scientific research to human health.

## Conclusion

The international community has a range of options which it could select for the governance of human genetic resources. Until it does so, it appears that they will, by default, fall under free access, privileging the interests of certain groups over others. This is not appropriate for resources which have a key role in combating global disease threats. Adoption of a common heritage of mankind approach to ownership/control of human genetic resources will align well with the key practical goals for their international governance, and with principles that have been outlined by the international community in relation to the management of the human genome and human genetic data. By excluding human genetic resources from state sovereign rights, intellectual property rights and other forms of appropriation, the common heritage approach will facilitate the dissemination of scientific knowledge and data, and broad international participation in research. It will also mean that decisions on which priorities to pursue will be based on universal interests rather than national interests and commercial potential.

## Abbreviations

CBD: Convention on Biological Diversity
CHM: Common heritage of mankind
FAO: Food and Agriculture Organisation
GISRS: Global Influenza Surveillance and Response System
TRIPS: Trade Related Aspects of Intellectual Property Rights Agreement
UNCLOS: United Nations Convention on the Law of the Sea
UNESCO: United Nations Educational, Scientific and Cultural Organisation
UNGA: United Nations General Assembly
WHO: World Health Organisation
WIPO: World Intellectual Property Organisation
WTO: World Trade Organisation
WTO-TNC: World Trade Organisation-Trade Negotiations Committee

## Footnotes

1 This is not the only way in which these approaches might be characterised and categorised. There are also other potential approaches which could be adopted by the international community (e.g. public trusteeship as outlined by Sand [20], however the focus in the paper is on those approaches already used by the international community in genetic resources governance.

2 The terms ownership and control are distinct. They are both used in this paper because some of the international approaches to genetic resources exclude ownership, while others blend elements of ownership and control.

3 An explanation of such a contract is provided by the secretariat of the International Treaty on Plant Genetic Resources at http://www.planttreaty.org/content/what-standard-material-transfer-agreement-smta.

4 Article 27—Patentable Subject Matter—of the Agreement on Trade Related Aspects of Intellectual Property Rights (TRIPS) states that “patents shall be available for any inventions, whether products or processes, in all fields of technology, provided that they are new, involve an inventive step and are capable of industrial application”.

5 To illustrate how extensive these discussions are, they have received the following coverage in TRIPS Council meeting minutes within the last 2 years: paragraphs 9–65 in IP/C/M/76/Add.1 *Minutes of Meeting 11 June 2014*; paragraphs 22–114 in IP/C/M/75/Add.1 *Minutes of Meeting 25–26 February 2014*; paragraphs 8–66 in IP/C/M/74/Add.1 *Minutes of Meeting 10–11 October 2013*; paragraphs 1–72 in IP/C/M/73/Add.1 *Minutes of Meeting 11-12 June 2013*; paragraphs 5.1–5.50 in IP/C/M/72 *Minutes of Meeting 5–6 March 2013*; and paragraphs 23–97 in IP/C/M/71 *Minutes of Meeting 6–7 November 2012*. All of these documents are available through WTO Documents Online Search Facility. http://www.wto.org/english/res_e/res_e.htm.

6 It is worth noting that there is a distinction between a commons and a common heritage of mankind approach—which is one way of managing a commons area/commons resources. Commons may also be subject to free access approaches for example—which, as noted in the main text, is the case for marine genetic resources within the high seas.

7 There are several conceptions of CHM in the literature; these are generally consistent with Joyner’s model, although not all include the points about scientific research (see, for example, [20] and [16]). Literature on CHM mainly stems from the field of environmental law. Taylor and Stroud’s [22] book *Common Heritage of Mankind: A Bibliography of Legal Writing* is an excellent starting point for those wanting to further explore the topic.

8 Regardless of the approach taken to the ownership of genetic resources, this clause will apply because of existing legal obligations in the Biological Weapons Convention, which prohibit any non-peaceful use of biological materials. (The Chemical Weapons Convention will similarly apply where genetic resources are exploited in order to produce chemical agents.).

9 Such acts are, of course, subject to limitations from other areas of law—this does not give the right to extract genetic material from an individual without their consent, for example.

10 This point is not integral to Joyner’s outline of CHM. However, special attention to the needs of developing countries does appear in relation to the management of CHM resources (for example Article 140 of the UNCLOS states that where activities take place to the benefit of mankind in the ‘area’, particular consideration should be given to the interests and needs of developing countries) and frequently appears in international rules on genetic resources.

11 It is worth noting that the human genome and human genetic resources are distinct, and discussion on the human genome as a commons and application of CHM to the human genome have a narrower focus than human genetic resources.

12 It does this, for example, in its *International Code of Conduct for Plant Germplasm Collection and Transfer* (1993), and *Genebank Standards for Plant Genetic Resources for Food and Agriculture* (2013).

13 These include the Human Genome Project, the 1000 Genomes Project, and the Encyclopaedia of DNA Elements Project.
